# Xpert MTB/RIF Assay for the Diagnosis of Lymph Node Tuberculosis in Children: A Systematic Review and Meta-Analysis

**DOI:** 10.3390/jcm11154616

**Published:** 2022-08-08

**Authors:** Hao-Kai Chen, Rui-Si Liu, Yi-Xuan Wang, En-Xiang Quan, Yuan-Hua Liu, Xu-Guang Guo

**Affiliations:** 1 Department of Clinical Laboratory Medicine, The Third Affiliated Hospital of Guangzhou Medical University, Guangzhou 510150, China; 2 Department of Clinical Medicine, The Third Clinical School of Guangzhou Medical University, Guangzhou 511436, China; 3 Department of Medical Imageology, The Second Clinical School of Guangzhou Medical University, Guangzhou 511436, China; 4 Department of Clinical Medicine, Nanshan College of Guangzhou Medical University, Guangzhou 511436, China; 5 Guangdong Provincial Key Laboratory of Major Obstetric Diseases, The Third Affiliated Hospital of Guangzhou Medical University, Guangzhou 510150, China; 6 Key Laboratory of Reproduction and Genetics of Guangdong Higher Education Institutes, The Third Affiliated Hospital of Guangzhou Medical University, Guangzhou 510150, China; 7 Guangzhou Key Laboratory for Clinical Rapid Diagnosis and Early Warning of Infectious Diseases, KingMed School of Laboratory Medicine, Guangzhou Medical University, Guangzhou 511436, China

**Keywords:** Xpert MTB/RIF, lymph node tuberculosis, children, meta-analysis

## Abstract

**Background**: Lymph node tuberculosis (LNTB) is the leading type of extrapulmonary tuberculosis (EPTB) causing death in children. The Xpert MTB/RIF assay is a novel rapid test for the diagnosis of LNTB. Although previous evidence suggests that Xpert is reliably accurate in diagnosing EPTB in children, information is lacking for the specific type of LNTB in children. The aim of this study was to systematically assess the accuracy and reliability of Xpert for the diagnosis of LNTB in children. **Methods:** We systematically searched four databases, Embase, Cochrane Library, PubMed, and Web of Science, which extracted relevant data according to predefined inclusion and exclusion criteria. The data were analyzed by meta-Disc 1.4 and Stata 12.0 software to determine sensitivity, specificity, diagnostic odds ratio (DOR), etc. **Results:** A total of 646 samples from 8 studies were included in the analysis. The pooled sensitivity, specificity, negative likelihood ratio (NLR), positive likelihood ratio (PLR,) and combined diagnostic odds ratio (DOR) of Xpert for all samples were 0.79 (95% CI 0.70, 0.87), 0.90 (95% CI 0.86, 0.92), 0.29 (95% CI 0.19, 0.43), 7.20 (95% CI 3.32, 15.60), and 37.56 (95% CI 13.04, 108.15), respectively. The area under the curve (AUC) of the summary receiver operating characteristic (sROC) curve was 0.9050. **Conclusion:** Overall, Xpert showed moderate sensitivity and high specificity compared with culture in the diagnosis of LNTB in children. In addition, after analyzing the combined diagnostic odds ratio and positive LR, our study showed that Xpert has excellent diagnostic accuracy.

## 1. Introduction

Tuberculosis (TB), a major infectious disease, is a serious global public health threat and a leading cause of death among children worldwide [1]. According to the World Health Organization’s annual Global Tuberculosis Report (2020 ver.), there were approximately 10 million TB cases worldwide in 2019, of which 12% of the new cases and 16% of the 14 million deaths were in children. Extrapulmonary TB accounts for 20–30% of all TB cases [2]. The incidence of EPTB is significantly higher in children and HIV patients than in normal controls [3]. Any organ throughout the body can be infected by EPTB, but the lymph nodes and pleura have been by far the most general locations of extrapulmonary infection [4]. Due to the diagnostic feature of EPTB, its incidence rate in children may be underestimated. As a result, the rapid diagnosis of EPTB in children is currently the focus of research in this field [5]. In this paper, we focus on the rapid diagnosis of LNTB in children.

LNTB is the most common kind of EPTB in children [6]. The estimated prevalence of LNTB in developing countries is 28%, much higher than in developed countries, which imposes a greater burden on medical care in less economically developed regions [7]. The most common form of intrathoracic lymphadenopathy in children under 5 years of age is tuberculosis of the hilar lymph nodes [8]. Studies have shown that lymph nodes are ecological niches for the growth and persistence of Mycobacterium tuberculosis. In addition, despite the presence of an immune response to Mycobacterium tuberculosis infection in the lymph nodes, their killing capacity is poor [9]. Many cases have shown that co-infection with LNTB and TB is common, suggesting a potential risk of LNTB [10].

The clinical manifestations of LNTB depend on the location of the affected lymph nodes and the immune status of the patient. Common symptoms include chest pain, abdominal distension, hemoptysis, weight loss, fatigue, and cough [11,12]. Besides, there is a mass in the lymph gland and its connected tissues in TB, especially in the neck, which produces pain. The majority of early-treated cases have a good prognosis, with a small number of related complications [12]. The transmission of Mycobacterium TB is determined by the infectious agent, of which airborne transmission is the main form [13]. The risk of transmission is highest when the patient performs sneezing, talking, or coughing. The effect of transmission depends on the individual fitness of the transmitter and the recipient.

LNTB is commonly diagnosed by FNA cytology, smear microscopy, and Mycobacterial culture. The advantages of smear microscopy are the rapid results and the simplicity of the procedure. However, the disadvantage is the low sensitivity of microscopy in detecting Mycobacterium TB in fine needle aspirate (FNA) specimens [14]. The advantages of FNA cytology (FNAC) for diagnosing LNTB are the clear diagnostic criteria and the ease of operation and judgment, but the disadvantage is its low specificity [15]. FNAC is only based on cell morphology and cannot be used as a reliable diagnostic method for LNTB detection alone [16]. Mycobacterium TB culture is currently the gold standard for the diagnosis of LNTB. However, it is difficult to be widely implemented in resource-limited countries or regions due to its long turnaround time and the need to be performed in a BSL3 laboratory [17]. In addition, as common samples in culture, FNA samples are oligobacterial, which may increase turnaround time or decrease their sensitivity [18]. Therefore, assessing the accuracy of the Xpert rapid test for the diagnosis of LNTB in children is important for the prevention, diagnosis, and treatment of this disease.

The Xpert^®^ MTB/RIF assay (Xpert; Cepheid, Sunnyvale, CA, USA) is a kit-based PCR test for the rapid diagnosis of TB. The advantages of our use of Xpert are its high sensitivity and the minimal technical expertise required to diagnose TB and simultaneously detect rifampicin resistance within 2 h [19]. Xpert is considered to exhibit higher sensitivity and specificity than microbiological and cytological assays [19]. The limit of detection (LOD) of Xpert is approximately 116 colony-forming units (cfu) per mL, and its sensitivity is still lower than that of culture (LOD 1–10 cfu/mL) [20]. There is still a gap in comparison with the accuracy of culture assays. In addition, the cost of Xpert is slightly higher than that of conventional assays.

Currently, Xpert is accepted for the diagnosis of EPTB, and in 2014, the WHO recommended the use of Xpert for the detection of specific non-respiratory specimens from patients with suspected EPTB [21]. However, there are few systematic and comprehensive analyses of the diagnostic accuracy of Xpert for specific extra-pulmonary sites of TB in a specific population of children with LNTB. We systematically evaluated the diagnostic accuracy and feasibility of using Xpert to detect LNTB in children with a comprehensive search and the inclusion of articles in a meta-analysis. This study will provide new insights into the rapid detection of LNTB in children.

## 2. Methods

### 2.1. Study Design

We conducted this study from October 2021 to March 2022, during which time researchers systematically assessed the diagnostic accuracy of Xpert for LNTB in children.

### 2.2. Search Strategy and Sources

Four panelists used the keywords “lymph node tuberculosis” and “Xpert MTB/RIF” and “children” in the Embase, Cochrane Library, PubMed, and Web of Science databases. The year and geography of the articles were not restricted in the search. Relevant literature was then screened according to inclusion and exclusion criteria, and articles that met the requirements were finally included in the meta-analysis.

### 2.3. Inclusion and Exclusion Criteria

#### 2.3.1. Inclusion Criteria

The inclusion criteria were (1) the detection of LNTB in children using Xpert, (2) literature data sufficient to plot a four-compartment table, (3) English-language literature, (4) analysis of human specimens, (5) availability of a gold standard against which target instrument results are compared.

#### 2.3.2. Exclusion Criteria

The exclusion criteria were (1) population not matching or being restricted to children; (2) Xpert instrument not being used; (3) conference abstracts, meta-analyses, editorials, and pathology reports; (4) disease not match or the test not being for LNTB; (5) a lack of direct or indirect access to the tetrad data; (6) a lack of a gold standard or a comparison of the target instrument with the gold standard.

### 2.4. Data Extraction

In reading the articles included in the analysis, the four panelists were separated into two groups and independently extracted the data as well as the necessary information, including the first author of the article, the author’s country, the time of publication of the article, the source of the specimen, the study design, the gold standard, the test of the gold standard, the method of detection, the type of sample, the number of true positives (TP), false positives (FP), true negatives (TN), and false negatives (FN). Finally, the extracted data were collated into a four-compartment table. The results of the data from the two groups were compared, and any differences that arose were resolved through discussion.

### 2.5. Quality Assessment of Studies

We assessed the quality of all eligible literature using the Quality Assessment of Diagnostic Accuracy Studies (QUADAS-2) guidelines. QUADAS-2 is based on four elements: patient selection, index test, reference standard, and process and time, with 11 criteria. The 11 criteria were rated using “Y (yes)”, “N (no)”, and “UC (unclear)” to evaluate the risk of bias and applicability issues. The final quality-assessment results were summarized in an Excel spreadsheet, and the data were visualized using ReviewManager 5.2 statistical software.

### 2.6. Statistics and Analysis

We used meta-Disc1.4 to analyze the extracted four-compartment table data to determine the sensitivity, specificity, positive likelihood ratio (PLR), negative likelihood ratio (NLR), diagnostic odds ratio (DOR), and their corresponding 95% confidence intervals (CI). Summary receiver operating characteristic (sROC) curves were plotted, and the area under the line (AUC) was calculated. The heterogeneity of the included studies was analyzed by I2, and the results were meta-analyzed using a random effects model for this study. We then plotted funnel plots using Stata 12.0 to assess the degree of bias (*p* < 0.05 was considered publication bias), and linear regression models were used to identify symmetry in the funnel plots.

## 3. Results

### 3.1. Results of Included Studies

**Figure 1:**
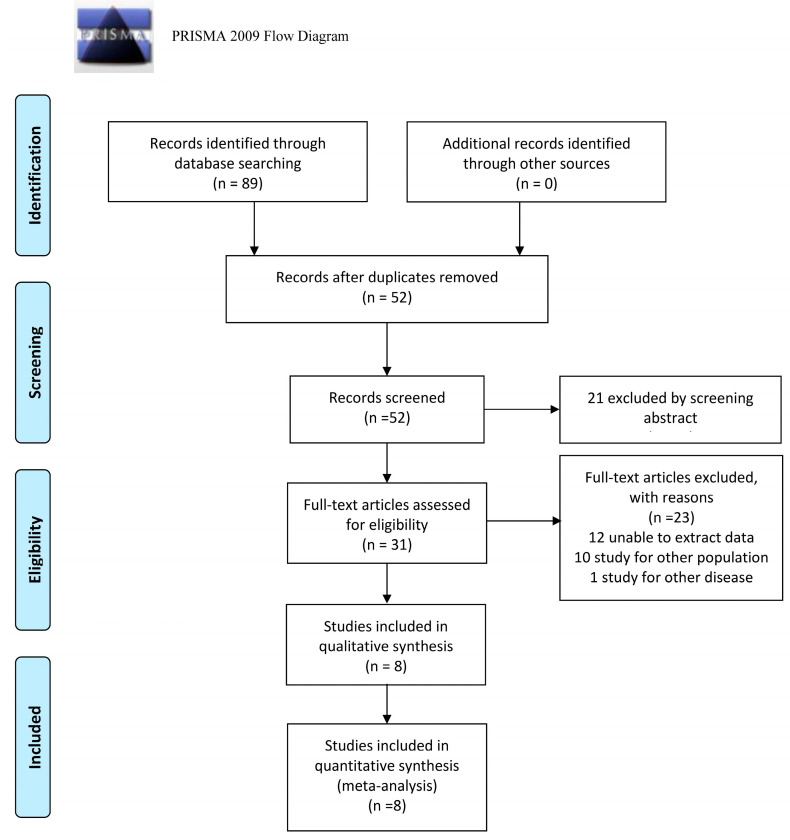
Flow chart of study identification and inclusion.

According to the previously developed search strategy, after a comprehensive database search, we found 27 articles in PubMed, 17 in Embase, 10 in the Cochrane Library, and 35 in Web of Science. Of the 89 articles, 37 were duplicates. After reviewing the titles and abstracts, 31 articles in total were entered into the full-text screening process. After eliminating 10 articles with noncompliant study populations, 1 article with noncompliant disease types and 12 articles with insufficient data, 8 articles [22,23,24,25,26,27,28,29] were finally included for full-text review and meta-analysis. The flow of the screening is shown in Figure 1.

### 3.2. Literature Characteristics

The eight articles included in the meta-analysis were published in 2014–2021, two of which were retrospective studies and the remaining six were prospective studies, and all were in English. Four studies were conducted in India and one each in Brazil, Tanzania, South Africa, and Thailand. The gold standard for inclusion in the studies was Mycobacterium TB culture. The samples analyzed in five of the eight articles were FNAB or lymph node FNA, and three were lymph node tissues from unknown sampling methods. The characteristics of these studies are summarized in Table 1.

### 3.3. Quality Assessment

**Figure 2:**
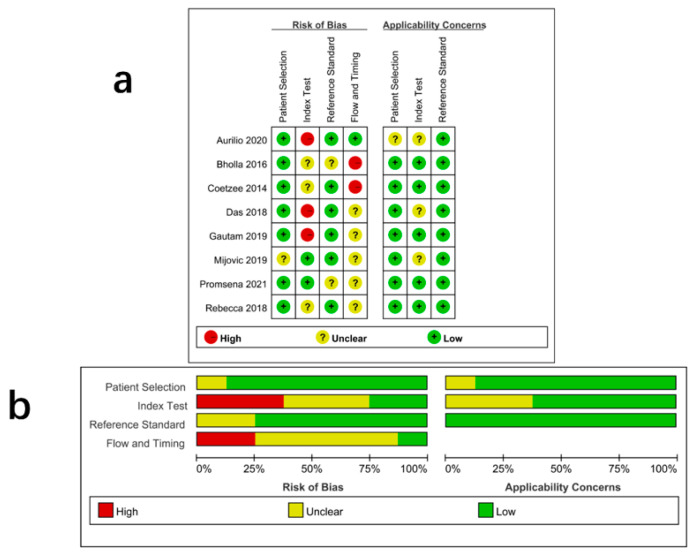
Quality evaluation of the included studies: (**a**) risk of bias and applicability concerns summary, (**b**) risk of bias and applicability concerns graph.

The quality of the eight included articles was assessed using Review Manager 5.3, and the results are shown in Figure 2a,b.

### 3.4. Summary Results

**Figure 3:**
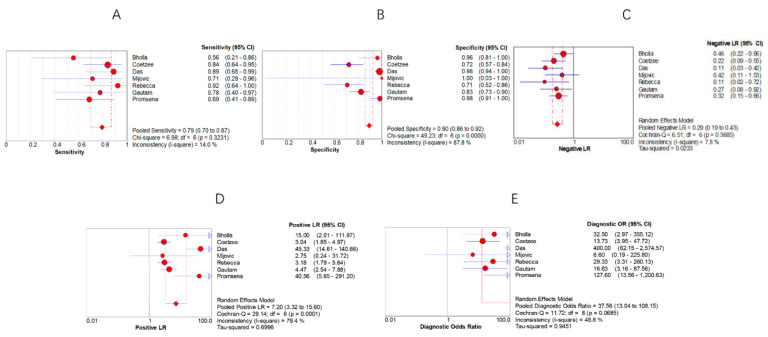
Forest plots of (**A**) sensitivity, (**B**) specificity, (**C**) negative likelihood ratio, (**D**) positive likelihood ratio, and (**E**) diagnostic odds ratio of Xpert for the diagnosis of LNTB in children.

**Figure 4:**
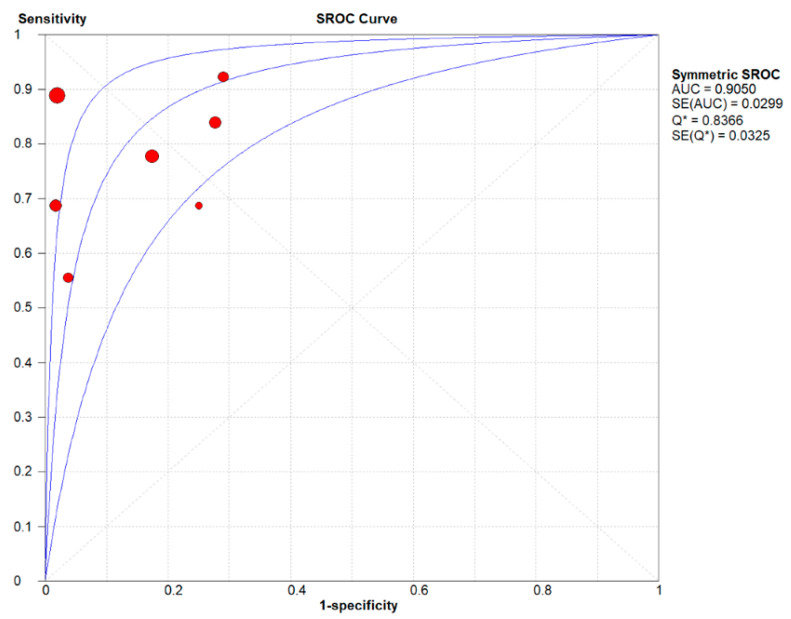
Summary receiver operating characteristic curve for the diagnosis of lymph node tuberculosis in children by Xpert.

For Xpert, the pooled sensitivities and specificities for all sample types were 0.79 (95% CI 0.70, 0.87) and 0.90 (95% CI 0.86, 0.92), respectively, with NLR and PLR values of 0.29 (95% CI 0.19, 0.43) and 7.20 (95% CI 3.32, 15.60). In addition, the value of DOR was 37.56 (95% CI 13.04, 108.15). The area under the curve of the sROC was 0.9050, and the Q-index was 0.8366. The I2 for sensitivity was 14.0%, and the I2 for specificity was 87.8%, indicating low heterogeneity in sensitivity and medium heterogeneity in specificity. In conclusion, our study shows that the instrument displays medium sensitivity and extremely high specificity, and its ability to identify uninfected individuals is more advantageous, making it a reliable rapid test for LNTB in children. The results of the above analysis are shown in Figure 3 and Figure 4.

### 3.5. Publication Bias

**Figure 5:**
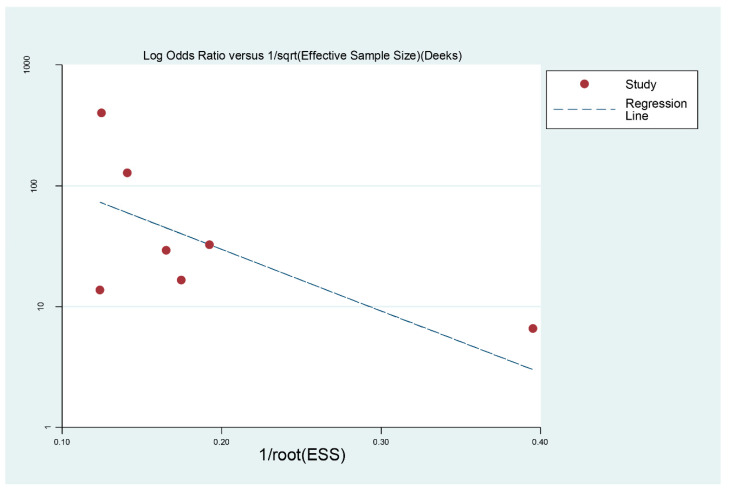
Deeks funnel plot to determine the publication bias of included articles.

Deeks’ funnel plots based on state 12.0 were produced to determine whether there was publication bias in the included studies. Our results showed a *p* value of 0.392, with little publication bias (Figure 5).

## 4. Discussion

Assessing the accuracy and feasibility of the Xpert rapid test for the diagnosis of LNTB in children is important for the prevention, diagnosis, and treatment of LNTB in children.

Currently, the main resistance to the large-scale application of Xpert is its unstable sensitivity and specificity. In this study, we searched four databases using stringent screening criteria, included eight articles, and performed a meta-analysis of them. After extracting data from 527 samples, we calculated pooled sensitivity, specificity, NLR, PLR, and DOR values for Xpert of 0.79 (95% CI 0.70, 0.87), 0.90 (95% CI 0.86, 0.92), 0.29 (95% CI 0.19, 0.43), 7.20 (95% CI 3.32, 15.60), and 37.56 (95% CI 13.04, 108.15), respectively. Moderate sensitivity indicates that negative test results with a high risk of positivity still require further testing to determine infection, and high specificity allows a positive result to be presumed to be a case of childhood LNTB. The PLRs of Xpert were all much greater than 10, and the DORs were all much greater than 1, indicating that they have high diagnostic accuracy. The SROC curve plotted by Xpert appears near the upper left corner and has an AUC value close to 1 (0.9050) and a Q-index of 0.8366. In summary, the results of the above instruments validate the fact that Xpert is a reliable and rapid-detection tool in the diagnosis of LNTB in children.

In this study, the I2 for the combined sensitivity of Xpert was 14.0%, and the I2 for the combined specificity was 87.8%, indicating low and almost negligible heterogeneity in sensitivity and high heterogeneity in specificity. To explore the source of heterogeneity, we examined forest plots and reviewed the data. In the Xpert specificity plot, Rebacca [25] and Gautam [27] et al. had low specificity and deviated significantly from the vertical line. A total of 2.9% of patients in Rebacca’s study had HIV coinfection, and all patients in Gautam’s study were unaware of HIV infection, suggesting that HIV coinfection with LNTB may be a source of concern due to its low specificity. In addition, the procedure, setting, and stability of the gold-standard test used in both studies may also be a source of heterogeneity.

Alexander W. Kay [30] and Young Seok Seo [31] published a systematic evaluation and meta-analysis on Xpert for the diagnosis of active TB or EPTB in children on 27 August, 2020, and on 6 January, 2020, respectively, and this study is an update of both studies. A more nuanced population and site of nodules is the hallmark of this study, and we searched for, included, and performed a systematic evaluation and meta-analysis of new studies on LNTB, a specific form of TB in children. After carefully examining the two aforementioned articles, we found the following differences by comparing them with the present study. (1) In terms of sample size, Alexander W. Kay included 318 samples, and Young Seok Seo included 277 samples, for a total of 527 samples being considered in the present study. After comparing the publication dates of the included studies, we found that a total of four newly published articles (Mijovic [26], Gautam [27], Aurilio [28], Promsena [29]) were included in this study, adding 369 samples to the analysis. The larger sample size means that the assessment of the diagnostic accuracy of Xpert is more convincing, and the inclusion of newly published articles is more indicative of the diagnostic accuracy of Xpert over recent time periods. (2) In terms of sensitivity and specificity, Alexander W. Kay achieved results of 0.904 and 0.898, and Young Seok Seo achieved results of 0.80 and 0.94. The pooled sensitivity value of 0.79 obtained in this study is slightly smaller than the results of the two articles mentioned above. The pooled specificity of 0.90 was slightly higher than that of Alexander W. Kay’s results but lower than that of Young Seok Seo’s results. The difference may be due to the difference in sample size, and we believe that the data calculated in this study are more reliable due to the inclusion of a larger sample size. (3) In terms of the analysis of the data, Alexander W. Kay and Young Seok Seo’s study only analyzed sensitivity, specificity, and sROC. The present study adds to theirs by analyzing NLR, PLR, and the combined DOR, thus making the results of the meta-analysis more accurate and convincing. In conclusion, this study complements and refines the meta-analysis on the diagnosis of LNTB in children with Xpert and provides a more adequate evidence-based medical basis for the widespread clinical use of Xpert.

However, there are still some limitations to this study. First, we only searched four databases for articles published before October 2021 and could not ensure that all articles were involved in the analysis. Second, although the sample size included in this study was much larger than previous studies of the same type, the number of articles was limited to eight, and further research on the diagnostic accuracy of the tool is therefore warranted. Third, the heterogeneity of the articles included in this study was not high, 87.8%, in terms of specificity, and the exact source of the heterogeneity is ambiguous. This may have swayed the reliability of the results to some degree.

## 5. Summary

The results of the above meta-analysis prove that Xpert is a reliable method for diagnosing LNTB in children and exhibits moderate sensitivity and high specificity. More experimental and clinical studies in different settings are still needed in the future to support the widespread clinical use of Xpert.

## Figures and Tables

**Table 1:** Characteristics of the included studies (*n*  =  8).

NO.	Author	Year	Study Design	Country	Source of Specimens	Gold Standard	Type of Apecimens	TP	FP	FN	TN
1	Coetzee [22]	2014	Prospective	South Africa	72 clinical specimens	Culture	fine needle aspiration biopsy (FNAB)	21	13	4	34
2	Bholla [23]	2016	Prospective	Tanzania	36 clinical specimens	Culture	fine needle aspirates of lymph nodes	5	1	4	26
3	Das [24]	2018	Prospective	India	171 clinical specimens	Culture	gastric aspirates, cerebrospinal fluids, induced sputum and lymph node aspirates	16	3	2	150
4	Rebecca [25]	2018	Retrospective	India	44 clinical specimens	Culture	extrapulmonary specimens	12	9	1	22
5	Mijovic [26]	2019	Prospective	India	8 clinical specimens	Culture	respiratory and extrapulmonary specimens	5	0	2	1
6	Gautam [27]	2019	Prospective	India	101 clinical specimens	Culture	fine-needle cytological aspirates	7	16	2	76
7	Aurilio [28]	2020	Retrospective	Brazil	20 clinical specimens	Culture	Cervical LNTB, Supraclavicular LNTB, Axillar LNTB, Inguinal LNTB	5	0	4	11
8	Promsena [29]	2021	Prospective	Thailand	75 clinical specimens	Culture	lymph nodes tissue from fine-needle aspiration or biopsy	11	1	5	58

## Data Availability

All data generated or analyzed during this study are included in this published article.

## Abbreviations

Xpert: Xpert MTB/RIF; LNTB: lymph node tuberculosis; EPTB: extrapulmonary tuberculosis; TB: tuberculosis; PLR: positive likelihood ratio; NLR: negative likelihood ratio; DOR: diagnostic odds ratio; sROC: summary receiver operating characteristic.
